# Computational tools for inversion and uncertainty estimation in respirometry

**DOI:** 10.1371/journal.pone.0251926

**Published:** 2021-05-21

**Authors:** Taewon Cho, Hodjat Pendar, Julianne Chung

**Affiliations:** 1 Department of Mathematics, Virginia Tech, Blacksburg, VA, United States of America; 2 Department of Biomedical Engineering and Mechanics, Virginia Tech, Blacksburg, VA, United States of America; 3 Computational Modeling and Data Analytics Division, Academy of Integrated Science, Virginia Tech, Blacksburg, VA, United States of America; Universidad Rey Juan Carlos, SPAIN

## Abstract

In many physiological systems, real-time endogeneous and exogenous signals in living organisms provide critical information and interpretations of physiological functions; however, these signals or variables of interest are not directly accessible and must be estimated from noisy, measured signals. In this paper, we study an inverse problem of recovering gas exchange signals of animals placed in a flow-through respirometry chamber from measured gas concentrations. For large-scale experiments (e.g., long scans with high sampling rate) that have many uncertainties (e.g., noise in the observations or an unknown impulse response function), this is a computationally challenging inverse problem. We first describe various computational tools that can be used for respirometry reconstruction and uncertainty quantification when the impulse response function is known. Then, we address the more challenging problem where the impulse response function is not known or only partially known. We describe nonlinear optimization methods for reconstruction, where both the unknown model parameters and the unknown signal are reconstructed simultaneously. Numerical experiments show the benefits and potential impacts of these methods in respirometry.

## Introduction

The overarching goal of this work is to develop practical mathematical and computational tools to advance reconstruction methodologies for the inverse problem of recovering signals in physiological systems from flow-through respirometry chambers. In many areas of biology and biomechanics, signals of interest cannot be measured directly, but instead must be estimated from indirect, noisy observations. For example, rates of oxygen consumption and CO_2_ production are important for measuring energy expenditure associated with physiological phenomena and can promote understandings of energy regulation systems, but recovering such information generally requires indirect calorimetry using respiration chambers.

In flow-through respirometry systems, the goal is to determine the pattern of real instantaneous gas exchange of an animal that is put in a chamber. Air is pumped through the chamber and continuously mixes with the CO_2_ and water vapor produced by the animal. Then, air that flows out of the chamber is brought to a gas analyzer that measures patterns of gas concentration. However, during this process the metabolic signals get distorted due to the washout kinetics. The recorded signal in the gas analyzer is a convolution of the true/instantaneous signal and the impulse response of the system which contains all characteristics of the respirometry system. The instantaneous signals of interest can only be obtained by solving so-called “input estimation” or “inverse” problems [1–3]. This problem like any other deconvolution or input estimation problem is inherently ill-posed. Finding the instantaneous signal is particularly important if we study the synchrony between the metabolic signals and other physiological measurements such as locomotion, food or drug consumption, or circadian rhythms.

For problems where the impulse response function of the system is known, the inverse respirometry reconstruction problem can be formulated as a *linear* inverse problem that resembles the widely-studied problem of deconvolution. Since it is well known that the respirometry problem is ill-posed, which means that small errors or measurement noise in the data may and often do lead to large errors in the reconstruction, regularization must be used. Some previous works that use Tikhonov regularization to solve the linear respirometry reconstruction problem include [3–5], among others. Since the forward model used in these regularized deconvolution methods is defined by the choice of the impulse response function, using an inaccurate impulse response function (e.g., one that is estimated experimentally) can result in significant degradation of the reconstruction accuracy. In flow through respirometry systems the pattern of the impulse response depends on the volume of the chamber, flow rate, size of the tubes between the chamber and gas analyzer, and even the size and location of the specimen in the chamber. Thus, it is important to consider methods that can either reconstruct the impulse response function or improve on a given impulse response function, while simultaneously reconstructing the desired signal. This problem is highly nonlinear and thus significantly more challenging to solve due to non-uniqueness of the solution. Indeed, joint reconstruction of the impulse response function and the physiological signal remains an open problem in the field of respirometry [6, 7].

In this paper, we consider computational methods for both the linear and nonlinear respirometry reconstruction problems, with a particular emphasis on large-scale problems. We begin by describing the underlying mathematical model. Let $h\in R^{n}$ define the impulse response function and let $x\in R^{n}$ contain the desired signal. Let $z=[\begin{array}{c} x\\ h \end{array}]\in R^{2n}$, then the observed signal contained in $b\in R^{n}$ can be modeled as,

$$\begin{array}{c} b=f(z)+\epsilon \;\text{with}\;f(z)=H(h)x, \end{array}$$ (1)

where $H\left(\cdot \right):R^{n}\to R^{n\times n}$ models the forward evolution process, and $\epsilon \in R^{n}$ represents noise or measurement errors. A common assumption is that the noise is independent and identically distributed from a Gaussian distribution with zero mean and variance *σ*^−2^, i.e., $\epsilon ∼N(0,\sigma^{-2}I)$. For a given **h**, the respirometry forward model can be represented with matrix **H**(**h**), which is highly structured. Specific details regarding **h** and **H** will be provided (see Mathematical Problem Set-up). Given **b** and **H**(·) the goal of the nonlinear (blind) respirometry problem is to reconstruct **z** (i.e., both **h** and **x**). Oftentimes, (1) is referred to as a *separable* nonlinear inverse problem. Notice that if **h** is fixed, then we have a linear inverse problem.

There are many computational challenges to solving respirometry problems. First, due to ill-posedness, an appropriate choice of regularization should be incorporated for stable solution computation, and this goes hand-in-hand with the challenging task of selecting a suitable regularization parameter. More specifically, for classic variational regularization of the linear respirometry problem, solution approximations are obtained by solving optimization problems of the form,

$$\begin{array}{c} \min_{x}\frac{1}{2}{∥Hx-b∥}_{2}^{2}+\frac{\lambda_{p}}{2}\Omega \left(x\right) \end{array}$$ (2)

where λ_*p*_ > 0 is a regularization parameter and $\Omega \left(\cdot \right):R^{n}\to R$ is a regularization functional determined by the choice of the prior. Previous studies on respirometry reconstruction employ standard Tikhonov regularization where $\Omega \left(x\right)={∥x∥}_{2}^{2}$, and practitioners manually tune the regularization parameter λ_*p*_. Selecting a suitable regularization parameter involves finding a good balance between introducing bias in the solution and preserving fidelity to the system and the observed data. This can be an expensive and time consuming task that requires multiple solves for various parameter choices [3–5]. Second, iterative methods provide an efficient approach to handle very large problems (e.g., large signals with many unknown parameters), but preconditioning techniques are needed to accelerate convergence and these preconditioners need to be tailored to the structure of matrix **H**. Third, it may be desirable to go beyond obtaining reconstructions to also provide uncertainty estimates for reconstructions, but this process often requires many expensive solves. The fourth, and most difficult, challenge is that methods need to be developed to handle nonlinearity in the problem (e.g., when the impulse response function contains errors or uncertainty). Due to difficulties of the nonlinear problem, previous respirometry studies do not formally consider this scenario. In this paper, we describe various approaches to address these challenges.

### Overview of contributions

For the linear respirometry problem, we present a Bayesian formulation of the inverse problem, where the unknown signal is modeled as a random variable with some probability distribution representing the uncertainties in the parameters. By adopting a Bayesian framework, we can not only compute reconstructions but also perform uncertainty quantification. We also compare different types of regularization techniques for solving the linear problem, and we propose efficient structure-exploiting preconditioners for accelerating iterative methods when applied to very large problems. These preconditioners are tailored to the respirometry problem since they exploit special structure in the model matrices. Then, we address the significantly more challenging problem of nonlinear respirometry reconstruction. We describe efficient nonlinear optimization methods to compute an approximation of **h** and **x** simultaneously, for scenarios where the impulse response function is not known but its support and delay are provided or can be estimated. In particular, we show that an alternating optimization method can be used with appropriate constraints (e.g., sparsity) to update both sets of parameters efficiently. To summarize, the novelty of this work is two-fold: First, for the linear respirometry problem, we provide a robust set of computational tools for solving the inverse problem (2). Contrary to existing respirometry studies where Tikhonov regularization is used, we describe methods that can include different regularizers, can accelerate iterate methods for large-scale problems, can automatically select regularization parameters, and can provide quantification of solution uncertainties. Second, for the nonlinear respirometry problem which has not been previously considered for respirometry, we propose computational methods for joint estimation of **h** and **x**. These methods distinguish the proposed work from existing studies on respirometry reconstruction.

An outline of the paper is as follows. We begin with a description of the mathematical set-up for the respirometry problem. We describe a Bayesian formulation of the linear respirometry problem and describe various tools for regularization and uncertainty quantification. We propose preconditioners for accelerating iterative methods. Then, we describe nonlinear optimization methods that can be used for nonlinear respirometry reconstruction. Numerical results for simulated and real respirometry data are provided to demonstrate the performance and potential of our proposed approaches.

### Mathematical problem set-up

We begin with a mathematical description of the forward model underlying respirometry. In a continuous input estimation scenario, we assume that the system is linear and time-invariant, such that the output signal can be written as a convolution of the instantaneous input signal and the impulse response function of the system. More precisely, the output of the system at time *t* is given by

$$b\left(t\right)=\int_{0}^{t}h\left(t-\tau \right)x\left(\tau \right)d\tau$$

where *x*(*τ*) describes the state of the system at time *τ* and *h* is the impulse response function. In a discrete formulation, we take observations at uniform time points 0 = *t*_0_ < *t*_1_ < … < *t*_*n*_ < ∞ denoted as

$$b_{k}=\sum_{i=0}^{k-1}h\left(t_{k}-t_{i}\right)x_{i}\delta t+\epsilon_{k},\;\text{for}\;k=1,\ldots ,n$$

where *b*_*k*_ and *x*_*k*_ describe the output and input signals respectively at time *t*_*k*_, *δt* = *t*_*k*+1_ − *t*_*k*_ is the sampling interval, and $\epsilon_{k}∼N\left(0,\sigma^{-2}\right)$ represent errors in the data. In matrix notation, we have the discrete respirometry problem,

$$\begin{array}{c} b=Hx+\epsilon , \end{array}$$ (3)

where

$$b=[\begin{array}{c} b_{1}\\ b_{2}\\ \vdots \\ b_{n} \end{array}],\;x=[\begin{array}{c} x_{0}\\ x_{1}\\ \vdots \\ x_{n-1} \end{array}],\;\epsilon =[\begin{array}{c} \epsilon_{1}\\ \epsilon_{2}\\ \vdots \\ \epsilon_{n} \end{array}],\;\text{and}\;H=\delta t[\begin{array}{cccc} h(\delta t) & 0 & \cdots & 0\\ h(2\delta t) & h(\delta t) & \ddots & \vdots \\ \vdots & \ddots & \ddots & 0\\ h(n\delta t) & \cdots & h(2\delta t) & h(\delta t) \end{array}].$$

Notice that if we let $h=\delta t[\begin{array}{c} h(\delta t)\\ \vdots \\ h(n\delta t) \end{array}]\in R^{n}$ be the discretized impulse response function, then **H** = **H**(**h**) is a lower-triangular Toeplitz matrix with **h** as the first column and we get a problem of the form (1).

For most respirometry problems, the impulse response function is not known in advance, but must be estimated experimentally. In practice, a CO_2_ pulse is injected into an empty chamber for a short time (e.g., 0.5 seconds) and the normalized recorded output serves as the impulse response function. Due to various experimental errors and imperfections, this process may result in an imprecise estimate of the impulse response function. Nevertheless, this is the standard process used in practice. One could consider using blind deconvolution methods to solve for **h** and **x** simultaneously given **b**, but this is a severely ill-posed problem where the main challenge is the existence of many local minimizers. Furthermore, the number of unknown variables doubles (i.e., 2*n* total unknowns in **x** and **h**). We will assume that partial information about the impulse response function is available and consider the so-called *semi-blind deconvolution* problem. More specifically, we assume that the delay and the support of the impulse response function are known. That is, let $s\in Z^{+}$ denote the support and $d\in Z^{+}$ represent the delay, then we assume that **h** has the form

$$\begin{array}{c} h={\left[\;\underset{d}{\underset{︸}{0\;\cdots \;0}}\;{\tilde{h}}^{⊤}\;\underset{n-d-s}{\underset{︸}{0\;\cdots \;0}}\;\right]}^{⊤}\;\text{where}\;\tilde{h}=\delta t{\left[\;h_{d+1}\;\cdots \;h_{d+s}\;\right]}^{⊤}\in R^{s} \end{array}$$

has nonzero elements and *h*_*k*_ = *h*(*kδt*). With these minor assumptions on the impulse response function, the nonlinear problem (1) reduces to smaller system given by,

$$\begin{array}{c} \tilde{b}=\tilde{H}\left(\tilde{h}\right)\tilde{x}+\tilde{\epsilon } \end{array}$$ (4)

where $b={\left[\;\underset{d}{\underset{︸}{{\bar{b}}^{⊤}}}\;{\tilde{b}}^{⊤}\;\right]}^{⊤}$ and $x={\left[{\tilde{x}}^{⊤}\;\underset{d}{\underset{︸}{\bar{x}}}\;\right]}^{⊤}\in R^{n-d}$ with $\tilde{b},\tilde{x},\tilde{e}\in R^{n-d}$, $\tilde{H}\left(\cdot \right):R^{s}\to R^{\left(n-d)\times (n-d\right)}$ with $\tilde{H}\left(\tilde{h}\right)$ being a lower-triangular Toeplitz matrix with ${\left[{\tilde{h}}^{⊤}\;\underset{n-d-s}{\underset{︸}{0\;\ldots \;0}}\;\right]}^{⊤}$ as the first column. The time response of a chamber is roughly about *V*/*F*, where *F* is the air inflow rate and *V* is the volume of the chamber [1, 4]. If there is no information about the support, we can assume it to be approximately 3 to 5 times the time response.

**Fig 1:**
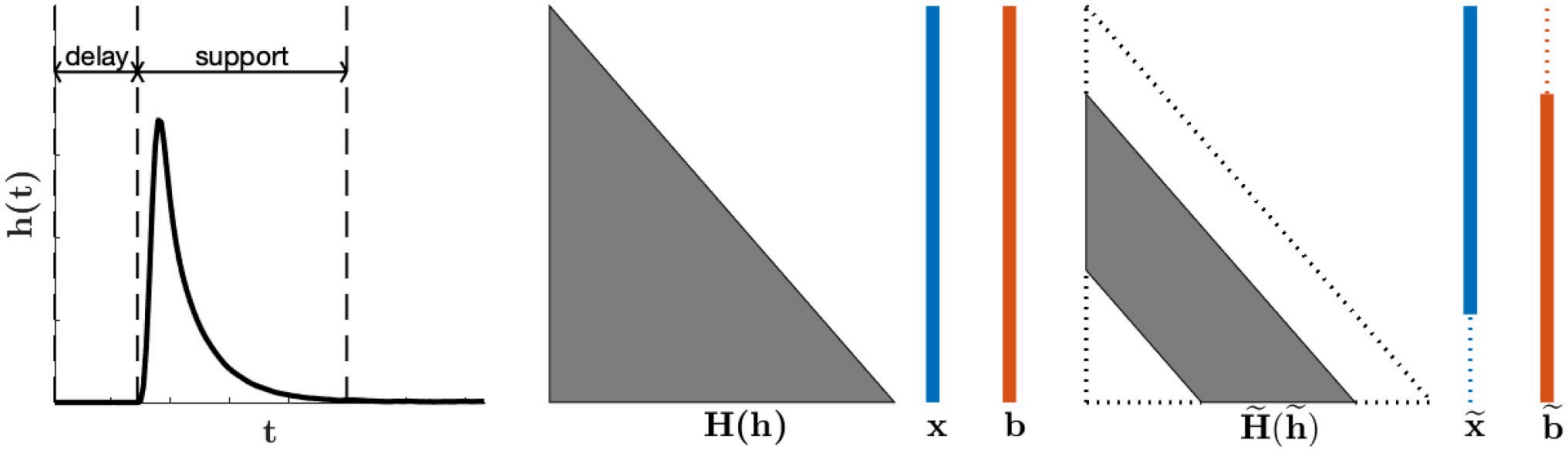
Illustration of the delay and support of the impulse response function *h*(*t*) used in respirometry. The middle and right plots demonstrate the change in structure from the original system in (1) to the reduced system in (4) that occurs due to the inclusion of delay and support assumptions.

An example of the delay and support of an impulse response function used in respirometry is provided in Fig 1, along with an illustration of the impact on the resulting system due to the delay and support. Notice that since **x** is replaced by $\tilde{x}$ in the reduced system, the tail of **x** is not being reconstructed. However, this is not a significant loss since it is common practice to ignore the final points of the reconstruction even in the non-blind case. The physical reason is that the released CO_2_ from the animal at the end of the experiment does not completely show up in our observed measurements since we have stopped recording before those CO_2_ particles leave the chamber and reach the gas analyzer.

We close this section with a few remarks. First of all, regarding our assumption of knowledge of the delay and support of the impulse response function, we can typically obtain good estimates of these values from the experimental impulse response function or from the respirometry problem set-up. Second, contrary to previous respirometry reconstruction methods, we do not assume any functional form for $\tilde{h}$. Third, we will describe various techniques to solve the reduced nonlinear system, and numerical results show that reconstructions are not sensitive to the choice of these parameters.

## Materials and methods

### Respirometry with *known* impulse response function

In this section, we assume that **h** is fixed and focus on efficient computational tools for solving the linear respirometry reconstruction problem (3) and for performing subsequent uncertainty quantification. Since the linear reconstruction problem is ill-posed, some form of regularization must be included. We employ a Bayesian framework, which is a statistically robust way to include prior knowledge by treating **x** as a random variable. Furthermore, the Bayesian approach provides a natural framework for performing uncertainty quantification. Good overviews on Bayesian inverse problems, statistical inverse problems, and computational uncertainty quantification can be found here [8–10].

#### Regularization and uncertainty quantification

Consider the stochastic extension of (3),

B=HX+E

where *X*, *B* and *E* are random variables and **H** is deterministic. We assume that *X* and *E* are mutually independent and that the prior density function of *X* is given by *π*_prior_(**x**) and the conditional density function of *B* given *X* is given by *π*_like_(**b** ∣ **x**). Using Bayes’ Theorem, the posterior probability density function can be written as

$$\begin{array}{c} \pi_{\text{post}}\left(x|b\right)\;=\;\frac{\pi_{\text{like}}\left(b|x\right)\;\pi_{\text{prior}}\left(x\right)}{\pi (b)}, \end{array}$$ (5)

assuming the marginal density function *π*(**b**) ≠ 0. Given observations in **b**, the solution of the inverse problem in the Bayesian formulation is the posterior distribution (5). This is a main distinction from classical inverse problems, where the solution is a single point estimate. The Bayesian formulation provides a natural framework for computing a family of solutions (e.g., many point estimates) and for providing qualitative information about the solutions (e.g,. measures of solution uncertainty).

A key component of the Bayesian formulation is the choice of the prior distribution function *π*_prior_(**x**), which incorporates any knowledge about the solution **x** prior to data being collected. Various methods can be used to create the prior. For example, previous experiments and reconstructions can be used to determine general features or characteristics of the solution. Priors can be learned from training data [11, 12] or can reflect knowledge about expected smoothness properties. In this paper, we consider two priors: a Gaussian prior and a Laplace prior. In both cases, we assume that the observation error can be modeled as $\epsilon ∼N(0,\sigma^{-2}I)$, and thus the likelihood function can be written as

$$\begin{array}{c} \pi_{\text{like}}\left(b|x\right)\;\propto \;\exp(-\frac{\sigma^{2}}{2}{∥Hx-b∥}_{2}^{2}). \end{array}$$

We will see that a nice connection between the Bayesian and classical formulations for inverse problems is that various point estimators in the Bayesian framework coincide with classic regularized solutions that are obtained by solving optimization problems of the form (2).

*2-norm regularization*. Gaussian priors are commonly used, and these priors are defined by a known mean vector $\mu \in R^{n}$ and known symmetric positive definite covariance matrix $\hat{Q}$, i.e., $x∼N(\mu ,\lambda^{-2}\hat{Q})$. In this case, the prior density function is given by

$$\begin{array}{c} \pi_{\text{prior}}\left(x\right)\;\propto \;\exp(-\frac{\lambda^{2}}{2}{\left(x-\mu \right)}^{⊤}{\hat{Q}}^{-1}\left(x-\mu \right)). \end{array}$$

and the posterior density function *π*_post_ is a Gaussian distribution,

$$\begin{array}{c} \pi_{\text{post}}=N\left(x_{\text{MAP}},\Gamma_{\text{post}}\right), \end{array}$$ (6)

where

$$\begin{array}{c} x_{\text{MAP}}=\Gamma_{\text{post}}\left(\sigma^{2}H^{⊤}b+\lambda^{2}{\hat{Q}}^{-1}\mu \right) \end{array}$$ (7)

with

$$\begin{array}{c} \Gamma_{\text{post}}={\left(\sigma^{2}H^{⊤}H+\lambda^{2}{\hat{Q}}^{-1}\right)}^{-1}. \end{array}$$ (8)

Let ${\hat{Q}}^{-1}=Q^{⊤}Q$ be a symmetric factorization (e.g., a Cholesky or eigenvalue decomposition), then the MAP estimate is the solution to the following optimization problem,

$$\begin{array}{cc} x_{\text{MAP}} & =\underset{x}{arg min}\frac{\sigma^{2}}{2}{∥Hx-b∥}_{2}^{2}+\frac{\lambda^{2}}{2}{∥Q\left(x-\mu \right)∥}_{2}^{2} \end{array}$$ (9)

$$\begin{array}{cc} & =\underset{x}{arg min}\frac{1}{2}{∥Hx-b∥}_{2}^{2}+\frac{\lambda_{2}^{2}}{2}{∥Q\left(x-\mu \right)∥}_{2}^{2} \end{array}$$ (10)

which is commonly known as Tikhonov regularization where $\lambda_{2}=\frac{\lambda }{\sigma }$. Thus, the Tikhonov regularized solution is the point estimate that corresponds to the maximum value of the posterior density function, or equivalently the minimizer of its negative log. Since the posterior density function is Gaussian, variance estimates for the solution can be obtained by computing the diagonal entries of **Γ**_post_. Furthermore, samples from the posterior can be obtained using efficient Krylov subspace methods [13, 14].

Note that in the inverse problems community, **Q** is often referred to as the regularization matrix and is chosen to force smoothness of the desired solution. There are many choices for the regularization matrix **Q**. In respirometry common choices for **Q** include the identity matrix **Q** = **I** or a discretization of the derivative operator where **Q** is a lower triangular Toeplitz matrix with [1 − 2 1 0 … 0]^⊤^ as the first column or [1 − 1 0 … 0]^⊤^ as the first column [4].

In addition to the choice of the regularization operator, the regularization parameter λ_2_ must be selected. There are various techniques to determine λ_2_ such as the discrepancy principle (DP), the L-curve, and the generalized cross validation (GCV) method [15]. Contrary to the DP and L-curve, the GCV method does not require a prior estimate of the noise level to determine the regularization parameter λ_2_. This is an advantage of the GCV method; however, computing the GCV regularization parameter can get costly especially for large-scale problems [16]. An alternative regularization technique is to use an iterative method (e.g., the Golub-Kahan bidiagonalization) to project a large-scale linear problem onto small but growing subspaces and to solve the projected problem using standard regularization techniques [17]. These are so-called *hybrid methods*. In [18], an implementation called HyBR combines the Golub-Kahan bidiagonalization and a weighted GCV method to solve problem (10) for **Q** = **I** that is efficient and can select the regularization parameter λ_2_ automatically. Various generalized Krylov techniques have been developed to handle the general form Tikhonov problem where **Q** ≠ **I** [19, 20], and various works have explored hybrid methods that can efficiently handle Gaussian priors by working directly with $\hat{Q}$ [21] or by working with mixed Gaussian priors [11].

*1-norm regularization*. An alternative assumption to a Gaussian prior is a Laplace prior, where the signal is independent and identically Laplace distributed,

$$\begin{array}{c} x_{i}∼\text{Laplace}\left(0,\delta^{-1}\right),\;i=1,2,\ldots ,n \end{array}$$ (11)

where the probability density function for a Laplace distribution is given by $\pi \left(x\right)=\frac{\delta }{2}\exp\left(-\delta |x|\right)$ for *δ* > 0. Thus, using the assumption of independence, the prior corresponding to assumption (11) can be written as

$$\pi_{\text{prior}}\left(x\right)\;\propto {\;\exp(-\delta ∥x∥}_{1}),$$

where ‖⋅‖_1_ is the 1-norm of a vector. The posterior density function *π*_post_ is given by

$$\begin{array}{c} \pi_{\text{post}}\left(x|b\right)\propto \exp(-\frac{\sigma^{2}}{2}{∥Hx-b∥}_{2}^{2}-\delta {∥x∥}_{1}). \end{array}$$ (12)

Notice that the posterior is no longer Gaussian; however, we can use various tools to explore the posterior. The MAP estimate corresponds to the mode of the posterior distribution and is given by

$$\begin{array}{cc} x_{\text{MAP}} & =\underset{x}{arg max}\pi_{\text{post}}\left(x|b\right) \end{array}$$ (13)

$$\begin{array}{cc} & =\underset{x}{arg min}\frac{\sigma^{2}}{2}{∥Hx-b∥}_{2}^{2}+\delta {∥x∥}_{1} \end{array}$$ (14)

$$\begin{array}{cc} & =\underset{x}{arg min}\frac{1}{2}{∥Hx-b∥}_{2}^{2}+\frac{\lambda_{1}}{2}{∥x∥}_{1}, \end{array}$$ (15)

which is an *ℓ*_1_-regularized problem (2) with Ω(⋅) = ‖⋅‖_1_ and $\lambda_{1}=\frac{2\delta }{\sigma^{2}}$.

It is common to use regularization terms of the form Ω(**x**) = ∥**x**∥_1_ in signal and imaging processing, since these regularizers enforce sparsity in the desired parameters. The main computational difficulty with these regularizers is the absolute value, which has a discontinuous first derivative at zero, causing challenges for optimization algorithms. For small to medium size problems, it is well known that the problem can be reformulated as a quadratic programming problem, and standard optimization software packages can be used. However, the number of unknowns in the reformulated problem doubles, making this approach unrealistic for large-scale problems. A more computationally appealing approach is to solve *ℓ*_1_-regularized problems using the proximal gradient. That is, methods such as the Fast Iterative Shrinkage-Thresholding Algorithm (FISTA) [22] use iterative techniques to solve

$$\begin{array}{c} \min_{x}\frac{1}{2}{∥Hx-b∥}_{2}^{2}+\frac{\lambda_{1}}{2}{∥x∥}_{1}. \end{array}$$ (16)

A summary of FISTA with a constant step size is provided in Algorithm 1.

**Algorithm 1** FISTA with constant stepsize

Choose λ_1_.

Compute *L*, a Lipschitz constant of $\frac{1}{2}{∥Hx-b∥}_{2}^{2}$.

Set $y_{1}=x_{0}=0\in R^{n}$ and *t*_1_ = 1.

**for** *k* = 1, 2, … **do**

$x_{k}=\underset{x}{arg min}\frac{L}{2}{∥x-(y_{k}-\frac{1}{L}H^{⊤}\left(Hy_{k}-b\right))∥}_{2}^{2}+\lambda_{1}{∥x∥}_{1},$

$t_{k+1}=\frac{1+\sqrt{1+4t_{k}^{2}}}{2},$

$y_{k+1}=x_{k}+(\frac{t_{k}-1}{t_{k+1}})\left(x_{k}-x_{k-1}\right).$

**end for**

In addition to the choice of the regularization parameter λ_1_ that must be selected in advance, the Lipschitz constant *L* that depends on the maximum eigenvalue of **H**^⊤^ **H** must be estimated. It can be difficult to compute *L* when *n* is large, but an approach using backtracking was described in [22]. Similar to FISTA, the Sparse Reconstruction by Separable Approximation (SpaRSA) method [23] is an iterative method that can be used to solve (16), which uses a sequence of smooth approximations of the 1-norm. Although more general regularization terms can be included, SpaRSA requires more user-defined input parameters so we do not consider it here. Another class of methods for solving the *ℓ*_*p*_-regularized problem is based on flexible Krylov methods that use iterative techniques with flexible preconditioning within a hybrid framework to improve the solution subspace. Methods such as FLSQR-R can be used to solve *ℓ*_*p*_-regularized problems where 1 ≤ *p* < 2, see [24].

Various methods for solving the *ℓ*_1_-regularized problem can be used to approximate the MAP estimate, but subsequent uncertainty quantification for this case is significantly more challenging. Although the posterior (12) is not Gaussian, we can approximate the posterior with a Gaussian at the maximum a posterior (MAP) estimate using a linearization approach [25]. Another approach to efficiently obtain samples from the posterior in this case is to use a change of variables or transformation to turn a non-Gaussian distribution into a Gaussian one, as described in [26]. More specifically, the transformation is defined by

$$\begin{array}{c} x=g\left(z\right)≔{\left[\begin{array}{ccc} g_{1D}\left(z_{1}\right) & \cdots & g_{1D}\left(z_{n}\right) \end{array}\right]}^{⊤} \end{array}$$ (17)

where

$$\begin{array}{c} g_{1D}\left(z\right)=L^{-1}G\left(z\right) \end{array}$$ (18)

with L being the cumulative density function (cdf) of the Laplace distribution and G being the cdf of a Gaussian distribution. With this definition, z∼N(0,I) and the transformation **z** = *g*^−1^(**x**) generates

$$\begin{array}{c} p\left(z\right)=p\left(g\left(z\right)\right)|J_{g}\left(z\right)| \end{array}$$ (19)

where

$$\begin{array}{c} J_{g}\left(z\right)=\text{diag}\left(g_{1D}^{\prime }\left(z_{1}\right),\ldots ,g_{1D}^{\prime }\left(z_{n}\right)\right). \end{array}$$ (20)

From these transformations and from (12), we obtain

$$\begin{array}{c} \begin{array}{ccc} p(z|b) & \propto & \exp(-\frac{1}{2}{∥[\begin{array}{c} H(g(z))\\ \sqrt{\lambda_{1}}z \end{array}]-[\begin{array}{c} b\\ 0 \end{array}]∥}_{2}^{2}) \end{array} \end{array}$$ (21)

Hence, we can generate samples from **z** and transform these samples to get samples of *p*(**x**|**b**) via **x** = *g*(**z**). Although there are some known challenges with this approach, we found that it worked well for the respirometry reconstruction problem.

By following a Bayesian framework for inversion, we have established a natural framework not only for incorporating prior knowledge but also for quantifying solution uncertainties. In terms of software, IRTools [27] is a comprehensive package that contains many iterative regularization routines for solving inverse problems along with various test problems. To the best of our knowledge there is no unified software package for performing UQ for inverse problems. We point the interested reader to the following book and associated codes [10].

#### Accelerating iterative methods for signal reconstruction

In the previous section, we considered various regularization techniques for solving the linear respirometry reconstruction problem. Next, we focus on Tikhonov regularization, and we investigate efficient methods to accelerate the convergence of iterative methods when used to compute a solution,

$$\begin{array}{cc} x_{\text{Tik}} & =\underset{x}{arg min}\frac{1}{2}{∥Hx-b∥}_{2}^{2}+\frac{\lambda_{2}^{2}}{2}{∥Q(x-\mu )∥}_{2}^{2} \end{array}$$ (22)

$$\begin{array}{cc} & ={\left(H^{⊤}H+\lambda_{2}^{2}Q^{⊤}Q\right)}^{-1}\left(H^{⊤}b+\lambda_{2}^{2}Q^{⊤}Q\mu \right), \end{array}$$ (23)

where (23) comes from setting the gradient of the function in (22) equal to zero. For small problems, constructing the matrix and the solution in (23) is computationally feasible, and many of the previous works in respirometry reconstruction follow this approach. For example, Tikhonov methods described in [5] could be used here. However, more sophisticated iterative techniques should be used for large-scale problems.

Iterative methods, in particular Krylov subspace methods, are computationally attractive because each iteration only requires one matrix-vector-multiplication with **H** and perhaps **H**^⊤^ [28, 29]. Thus, the matrix representing the respirometry forward model never needs to be constructed, but instead can be accessed via operations or function evaluations. However, it is widely known, especially in the numerical linear algebra community, that preconditioning is a very important tool for accelerating convergence and improving the robustness of Krylov methods [30]. The basic idea of preconditioning is to modify the problem by improving the spectrum of the problem (so that eigenvalues or singular values of the preconditioned system are clustered around one and bounded away from zero), thereby accelerating the convergence of iterative methods.

For simplicity of presentation, we describe preconditioning techniques for the unregularized problem (i.e., λ_2_ = 0) and focus on developing a good preconditioner for the respirometry matrix **H** that can exploit the special structure of these matrices. We first describe the general idea underlying preconditioning and then describe how to apply preconditioning to the regularized problem.

Assume that we have a preconditioner $M\in R^{n\times n}$ such that **M**^−1^ ≈ **H**^−1^ and solving systems involving **M** can be done easily and quickly. Then rather than solve (22), consider solving the right-preconditioned problem,

$$\begin{array}{c} \min_{y}{∥HM^{-1}y-b∥}_{2}^{2}\;\text{where}\;y=Mx, \end{array}$$

or the left-preconditioned problem,

$$\begin{array}{c} \min_{x}{∥M^{-1}Hx-M^{-1}b∥}_{2}^{2} \end{array}$$

using an iterative method such as the conjugate gradient for least-squares (CGLS) method [31]. Notice that each iteration requires one matrix-vector multiplication with **M**^−1^ **H** and its transpose. Typical choices for **M** are based on incomplete matrix factorizations or multigrid methods [30]. However, for the respirometry problem, these approaches are not ideal for two main reasons. First, matrix **H** is large and construction of **H** is not possible. Instead, we access it via function evaluations. Second, **H** is severely ill-conditioned, so we would like to approximate a regularized pseudoinverse of **H** rather than **H**^−1^. Obtaining a good approximation of **H**^−1^ would result in very fast convergence to the undesired inverse solution.

For respirometry reconstruction problems, we propose various preconditioners that can be used to accelerate the convergence of iterative methods. Recall that **H** is a Toeplitz matrix with **h** as the first column. For large-scale problems, we have constructed an object class in MATLAB called convMatrix.m, where matrix-vector and matrix-transpose-vector operations with **H** are treated as function evaluations. In particular, convMatrix calls MATLAB’s conv function to do convolution and then extracts the appropriate signal length.

Next, we describe how to exploit the Toeplitz structure of **H** to build a good preconditioner for the respirometry problem. Since circulant matrices provide good approximations to Toeplitz matrices [32–35] and circulant matrices are diagonalized by the discrete Fourier transform, we propose to use a *circulant* matrix **M** such that the lower triangular part of **M** matches the lower triangular part of **H**. Then since **M** can be diagonalized by the discrete Fourier transform, we can write

$$\begin{array}{c} M=F^{*}\Theta F \end{array}$$ (24)

where **F** represents the Fourier transform and **Θ** is a diagonal matrix with eigenvalues computed as

theta=fft(h);

where **h** contains the impulse response function scaled by the sampling rate (i.e., this corresponds to the first column of **H**). Thus, we can apply the preconditioner to any vector **y** as

$$M^{-1}y=F^{*}\Theta^{-1}Fy,$$

which corresponds to the following commands in MATLAB

ifft(fft(y)./theta);.

Notice that since **M** is likely ill-conditioned, **Θ** has very small values on the diagonal which can result in erroneous computations. A small modification to the preconditioner can be done, where **Θ** is replaced with a diagonal matrix $\hat{\Theta }$ with better spectral properties (i.e., removing small eigenvalues of **Θ**). For simplicity, we can use a TSVD-like preconditioner where $M=F^{*}\hat{\Theta }F$ with diagonal entries of $\hat{\Theta }$ being

$$\begin{array}{c} {\hat{\theta }}_{i}=\{\begin{array}{cc} \theta_{i}, & \text{if}\;|\theta_{i}|\geq \tau \\ 1, & \text{else} \end{array} \end{array}$$ (25)

for some predetermined tolerance parameter *τ*. Because the preconditioner inherently includes regularization, we avoid the danger of the preconditioner inadvertently magnifying the noise before a solution can be computed. Similar to the previous discussion about avoiding the construction of **H**, we remark that construction of the preconditioner is also not advised. We have written an object class called precMatrix.m that can work with the preconditioner implicitly. The preconditioner for respirometry can be accessed using the MATLAB command: M = precMatrix(h,tau); where **h** contains the impulse response function without delay and *τ* is the tolerance parameter. We also describe an automatic approach to estimate *τ*. Consider the approximate problem **Mx** = **b**. Since the diagonalization of **M** is computable, we can use the GCV method to efficiently compute a regularization parameter or truncation tolerance for TSVD [15]. This truncation tolerance can be used to define the preconditioner. Furthermore, we remark that **M** corresponds to convolution with the same impulse response function, where periodic boundary conditions are assumed. Thus, if **H** corresponds to periodic boundary conditions and *τ* is the smallest singular value, then **M** = **H**.

**Fig 2:**
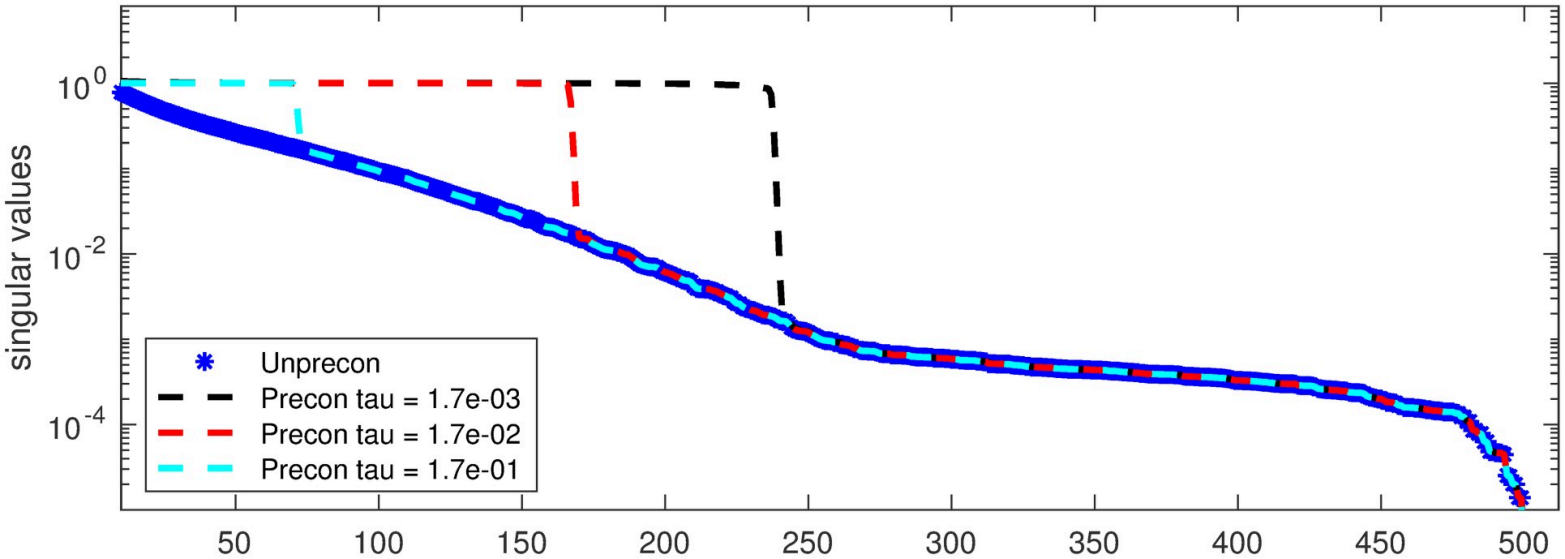
Spectrum of the unpreconditioned and preconditioned respirometry matrices for various choices of *τ*. Note the desirable clustering of the larger eigenvalues, which results in fast convergence of iterative methods.

For a small respirometry example, we provide in Fig 2 the spectrum of **H** along with the spectrum of the preconditioned system **M**^−1^ **H** for *τ* = 4.5 × 10^−2^, 9.0 × 10^−2^, and 1.8 × 10^−1^. The GCV selected parameter for this example was 9.0 × 10^−2^. Notice that the singular values for the preconditioned systems are clustered around 1. This is a very desirable property for the fast convergence of Krylov subspace methods [29]. However, the spectrum of the preconditioned system relies heavily on the choice of *τ*. For small values of *τ*, **M** clusters too many of the small singular values so that the preconditioned system will be very ill-posed. On the other hand, for larger values of *τ*, only a few singular values are clustered so more iterations would be required. If *τ* is greater than or equal to the largest singular value of **H**, then **M** = **I** and we have no preconditioning.

We have focused on developing preconditioners that exploit the structure of **H** and described how to use these preconditioners to accelerate iterative methods. If one wishes to use these preconditioners for solving regularized problems (e.g. (22)), then a simple extension can be made. That is, one can solve preconditioned problem,

$$\begin{array}{c} \min_{y}{∥HM^{-1}y-b∥}_{2}^{2}+\lambda_{2}^{2}{∥Q(M^{-1}y-\mu )∥}_{2}^{2}\;\;\text{where}\;\;y=Mx, \end{array}$$

or

$$\begin{array}{c} \min_{x}{∥M^{-1}Hx-M^{-1}b∥}_{2}^{2}+\lambda_{2}^{2}{∥Q(x-\mu )∥}_{2}^{2}. \end{array}$$

In general, proper preconditioning can be a very important, albeit delicate, task especially for inverse problems.

### Respirometry with *unknown* impulse response function

Thus far, we have focused on the linear respirometry problem where the impulse response function is assumed known. However, this is not true in realistic experiments, where the impulse response function must be estimated. Given measured respirometry data, estimating both the impulse response function and the unknown signal simultaneously is a very challenging problem. Nevertheless, there are various reasons why we may want to consider a joint estimation approach. First, the estimated impulse response function which is obtained using a short burst of CO_2_ in an empty chamber will likely contain errors. Second, although the respirometry systems is calibrated at construction, parameters may change over time and these changes are not accounted for without a full recalibration of the machine. Third, the impulse response depends on the size and location of the animal, which could change across experiments. For these and other reasons, we are interested in methods that can solve the nonlinear respirometry reconstruction problem. It is worth mentioning that we tried some off-the-shelf blind deconvolution methods such as MATLAB’s deconvblind function, but found that reconstructions were very poor; thus motivating us to consider alternative approaches.

The impulse response function, which models the reaction of the system to a very short unit impulse in a linear time-invariant system, is a key component of respirometry reconstruction. Conventional methods such as the Z-transform method described in [1] use an impulse response function defined by an exponential function, e.g., *h*(*t*) = *αe*^−*βt*^ where *α* and *β* are parameters defined by the flow rate and chamber volume. In [4], the authors experimentally showed that for many chambers and flow rates, the impulse response has the form *h*(*t*) = *αt*^*m*^ *e*^−*βt*^ where *α*, *m*, and *β* are parameters of the system. Although the parameters for the impulse response function must be estimated, numerical experiments showed that this function performed better than the exponential function. For the methods described in this section, we do not enforce a functional form for the impulse response function. Instead we impose other less restrictive constraints on the impulse response function, and develop computational methods for nonlinear respirometry reconstruction, where both the signal **x** and the impulse response function **h** can be estimated simultaneously from the data. The goal is to solve *nonlinear* optimization problem,

$$\begin{array}{c} \min_{x,h}{∥H\left(h\right)x-b∥}_{2}^{2}+\lambda_{p}\Omega \left(x\right)+\lambda_{h}{∥h∥}_{2}^{2}\;\text{s.t.}\;h\geq 0\;\text{and}\;\sum_{i=1}^{n}h_{i}\delta t=1 \end{array}$$ (26)

where λ_**h**_ is a regularization parameter for **h**. Compared to problem (2), we have a nonlinear model represented by **H**(**h**), and we have various additional constraints on **h**. These constraints include an additional Tikhonov regularization term for **h** to enforce smoothness, a nonnegativity constraint, and a mass preserving constraint to force the computed impulse response function to sum to 1. This last constraint corresponds to forcing the integral of the impulse response function to be 1 in the continuous framework.

**Fig 3:**
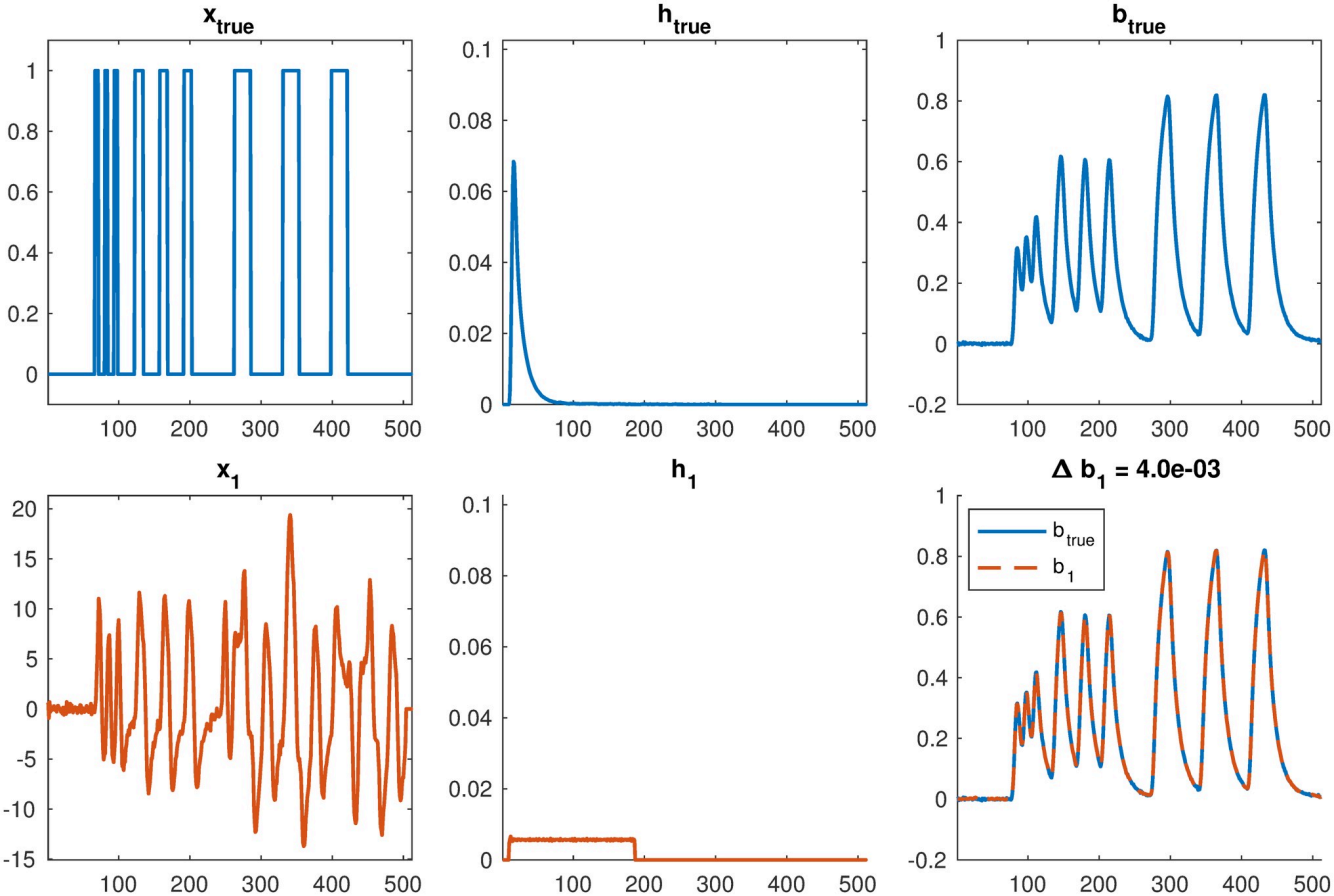
Illustration of the non-uniqueness problem in blind respirometry reconstruction. Both sets of parameters in **x** and **h** result in similar observed measurements in **b**. The result in the second row corresponds to using Tikhonov regularization for **x** and solving **h** using alternating optimization.

Before we describe computational methods to solve nonlinear constrained optimization problem (26), we provide an example to illustrate why solving the nonlinear blind reconstruction problem is significantly more difficult. The main concern is the existence of multiple minimizers. That is, without additional constraints, there are multiple solution pairs (**x**, **h**) that give small values of the data fit term in the objective function. The plots in Fig 3 show that under the convolution operation, two very different pairs (**x**, **h**) can result in nearly the same observation. Thus, methods for numerical optimization can easily get trapped in local minimizers. Including additional constraints can help with this problem. We observed that the choice of regularization for **x**, i.e., the choice of Ω(**x**), was important. In particular, reconstructions obtained using Tikhonov regularization in the nonlinear framework resulted in significantly smaller residual error norms, which negatively impacted the convergence. However, using Ω(**x**) = ∥**x**∥_1_ resulted in faster convergence and better reconstructions.

Next we describe a computationally efficient method to solve (26). First, following the model described in (4), we assume that the delay and support of **h** are known and reformulate problem (26) as

$$\begin{array}{c} \min_{\tilde{x},\tilde{h}}∥\tilde{H}\left(\tilde{h}\right)\tilde{x}-\tilde{b}{∥}_{2}^{2}+\lambda_{1}∥\tilde{x}{∥}_{1}+\lambda_{\tilde{h}}{∥\tilde{h}∥}_{2}^{2}\;\text{s.t.}\;\tilde{h}\geq 0\;\text{and}\;\sum_{i=d+1}^{d+s}h_{i}\delta t=1 \end{array}$$ (27)

where λ_1_, $\lambda_{\tilde{h}}$ are regularization parameters for $\tilde{x}$ and $\tilde{h}$ respectively. For large scale problems, matrix-vector multiplications $\tilde{H}\left(\tilde{h}\right)\tilde{x}$ are done via function evaluations so that $\tilde{H}$ is never constructed explicitly. Also note that $\tilde{h}$ and $\tilde{x}$ are exchangeable since

$$\begin{array}{c} \begin{array}{ccc} \tilde{H}\left(\tilde{h}\right)\tilde{x} & = & {\hat{H}}_{s}\left(\tilde{x}\right)\tilde{h} \end{array} \end{array}$$ (28)

where ${\hat{H}}_{s}\left(\tilde{x}\right)\in R^{(n-d)\times s}$ contains the first *s* columns of a lower-triangular Toeplitz matrix with $\tilde{x}$ as its first column. We will exploit this property in the described alternating optimization method.

Various nonlinear optimization methods can be used to solve problem (27) [36, 37]. A fully coupled approach would update all variables simultaneously, e.g., an inexact Newton method to solve for $\tilde{z}=[\begin{array}{c} \tilde{x}\\ \tilde{h} \end{array}]$. The main caveats of this approach are that derivatives are required and convergence can be slow. On the other hand, an alternating approach can be used to exploit the separability of the parameters in $\tilde{x}$ and $\tilde{h}$. That is, we alternate between fixing $\tilde{h}$ and optimizing over $\tilde{x}$, and fixing $\tilde{x}$ and optimizing over $\tilde{h}$. An alternating optimization method to solve (27) is provided in Algorithm 2. Notice that a key computational benefit of the alternating optimization approach for this problem is that by exploiting property (28), each optimization problem corresponds to solving a linear inverse problem.

**Algorithm 2** Alternating Optimization for Blind Respirometry

choose initial ${\tilde{h}}_{0}$ and tolerance tol

**for** *k* = 0, 1, 2, … **do**

${\tilde{x}}_{k}=\underset{\tilde{x}}{arg min}{∥\tilde{H}\left({\tilde{h}}_{k}\right)\tilde{x}-\tilde{b}∥}_{2}^{2}+\lambda_{1}{∥\tilde{x}∥}_{1}$

${\tilde{h}}_{k+1}=\underset{\tilde{h}}{arg min}{∥{\hat{H}}_{s}\left({\tilde{x}}_{k}\right)\tilde{h}-\tilde{b}∥}_{2}^{2}+\lambda_{\tilde{h}}{∥\tilde{h}∥}_{2}^{2}\;\text{s.t.}\;\tilde{h}\geq 0\;\text{and}\;\sum_{i=d+1}^{d+s}h_{i}\delta t=1$

$\tilde{r_{k}}=\tilde{H}\left({\tilde{h}}_{k}\right){\tilde{x}}_{k}-\tilde{b}$

 **if** $∥{\tilde{h}}_{k+1}-{\tilde{h}}_{k}{∥}_{2}<\text{tol}$ **or** ${∥{\tilde{r}}_{k}-{\tilde{r}}_{k-1}∥}_{2}<\text{tol}$ **then**

  **stop**

 **end if**

**end for**

In summary, we reformulated the blind respirometry reconstruction problem as a constrained nonlinear optimization problem, where the additional constraints are modest and reasonable. We assume that the delay and the support of the impulse response function are known, and we describe an alternating optimization method to estimate both the impulse response function and the instantaneous signal. In general, alternating optimization methods can be slow to converge but can have fast convergence if the initial guess is close to a minimizer. By exploiting structure in the problem, we have reduced the overall computational costs. We remark that for problems where the impulse response function can be parameterized using a few variables, a variable projection method may be used [38], but including additional constraints is not straightforward.

## Results and discussion

**Fig 4:**
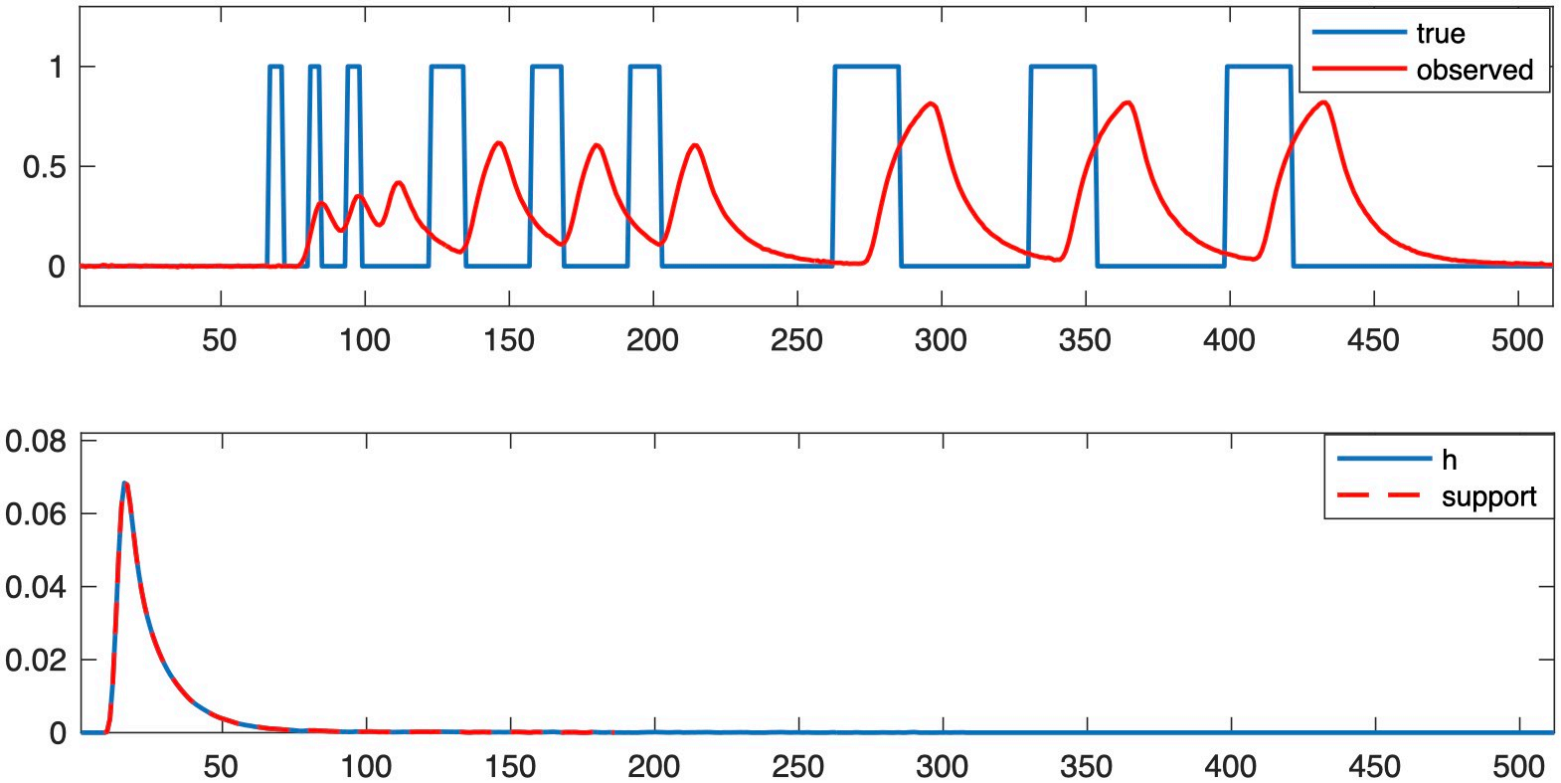
Simulated problem setup. The true signal and simulated observation with noise level 0.5% are provided in the top plot, and the impulse response function and its support are provided in the bottom plot.

In this section, we compare numerical optimization methods for different regularization functions and demonstrate the performance of the proposed preconditioners for the linear respirometry problem. We provide numerical results for uncertainty quantification for both Tikhonov and 1-norm regularizers. Then, we present results for a nonlinear respirometry reconstruction problem, where robustness of the proposed nonlinear optimization method is investigated. For the simulated dataset, we generate the measurements as in (1), where **x**_true_, **h** and **b** are provided in Fig 4 where the noise level is 0.5% and *n* = 512. We remark that real metabolic signals usually have slowly varying patterns. However, some species exhibit discontinuous cycles of ventilation with periods of little to no CO_2_ release [39, 40]. Here, to test the methods we choose **x**_true_ to be a series of rectangular pulses with various durations and frequencies. The rectangular pulses contain high frequency elements and recovering these signals is more challenging comparing to smooth patterns. For the linear respirometry results, we assume that we are given **h** and **b**, and we seek reconstructions of **x**_true_. For the nonlinear respirometry results, we assume that we are given **b** as well as the delay and support of **h**, and we seek reconstructions of **x**_true_ and **h**. In addition to the simulated studies, we provide a case study for experimental validation on real data for both the linear and nonlinear respirometry problems.

### Linear respirometry reconstruction

For the linear respirometry problem, we investigate reconstructions using a 2-norm and a 1-norm regularization term. We consider two Tikhonov regularized solutions. Tikhonov-**Q** incorporates a regularization matrix **Q**, which is a lower triangular Toeplitz matrix with [1 − 1 0 … 0]^⊤^ as the first column [5], and HyBR-I corresponds to a hybrid iterative projection method with regularization matrix **Q** = **I**. For the 1-norm penalty, we investigate a flexible hybrid iterative method called FLSQR-R [24] and FISTA as described in Algorithm 1.

For the regularization parameter λ, we use the optimal regularization parameter for Tikhonov-**Q**, which corresponds to minimizing the relative error between the reconstruction and the true signal. Both hybrid methods HyBR-I and FLSQR-R determine the regularization parameter automatically at each inner iteration using the weighted GCV method [18, 24]. For FISTA, the regularization parameter must be fixed in advance, and we set λ_1_ = 0.002.

**Fig 5:**
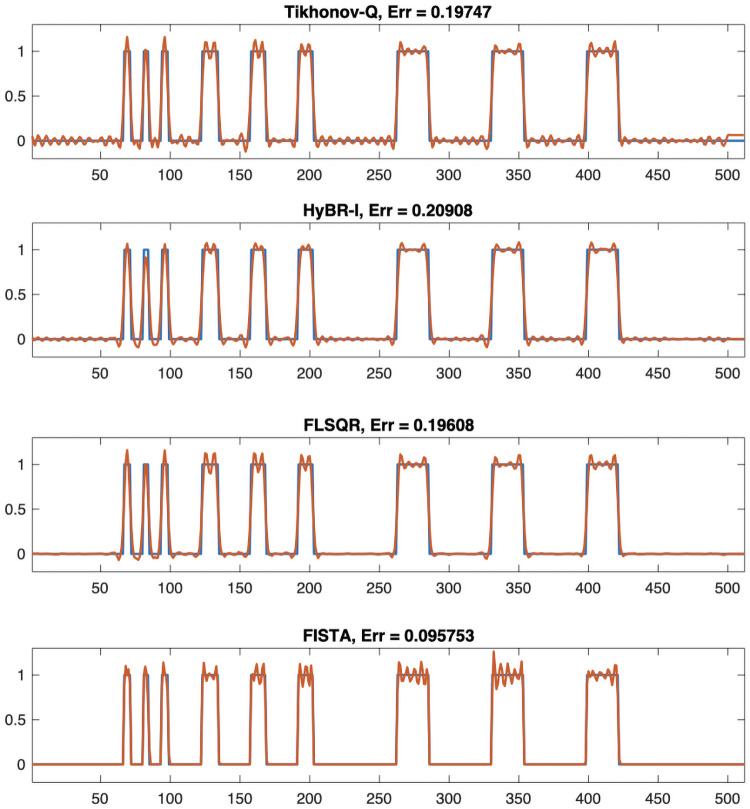
Reconstructions for linear respirometry reconstruction. Tikhonov-**Q** and HyBR-I reconstructions correspond to *ℓ*_2_ regularization, and FLSQR-R and FISTA reconstructions correspond to *ℓ*_1_ regularization. The true signal is provided in the blue line and the reconstructions are provided in red. Relative reconstruction error norms computed using the 2-norm are provided in the titles.

From the reconstructions in Fig 5, we observe that FLSQR-R and FISTA enforce sparsity in the reconstructions, and thus there are fewer artifacts. The FISTA reconstruction had the smallest relative reconstruction error norm among the considered methods, but this approach requires a good choice of the regularization parameter a priori, which requires time and careful tuning.

**Fig 6:**
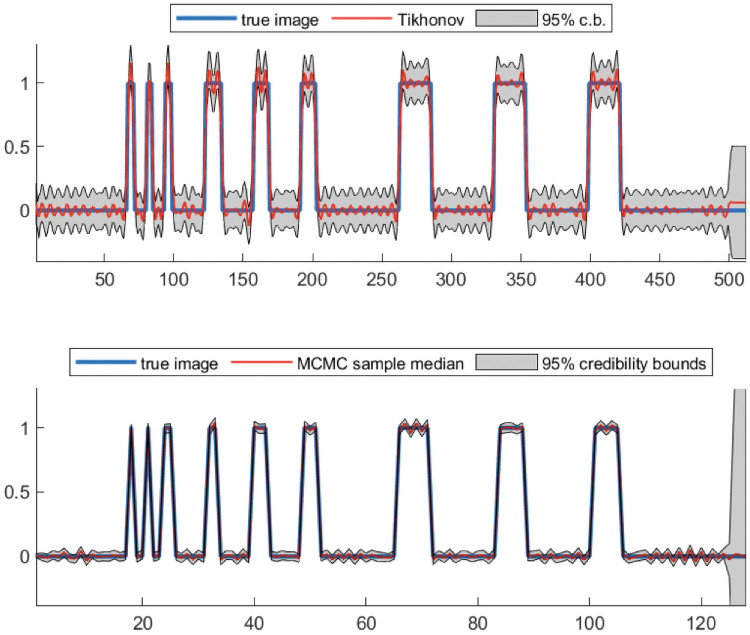
Uncertainty quantification for linear respirometry. The top plot contains the Tikhonov-**Q** solution with the 95% credibility bounds, and the bottom plot contains the sample median and 95% credibility bounds with 1000 samples corresponding to the Laplace prior.

Next we provide credibility bounds for reconstructions of the linear respirometry problem. In Fig 6 we provide the Tikhonov-**Q** reconstruction from Fig 5 with λ_2_ = 0.0114 along with the 95% credibility bounds. These 95% credibility bounds are computed from (8). Performing uncertainty quantification for Laplace priors (corresponding to the 1-norm) is a bit more difficult. We use the transformation described in [26] for Markov Chain Monte Carlo sampling. More specifically, we compute 1000 samples from the posterior distribution, using the approach described in the Matlab code OneDBlurHarr.m from Section 6.4 of [10]. Due to computational difficulties for large-scale problems, we reduce the signal size to *n* = 128. As expected, we observe larger variances at the tail of the signal due to the delay in **h**.

**Fig 7:**
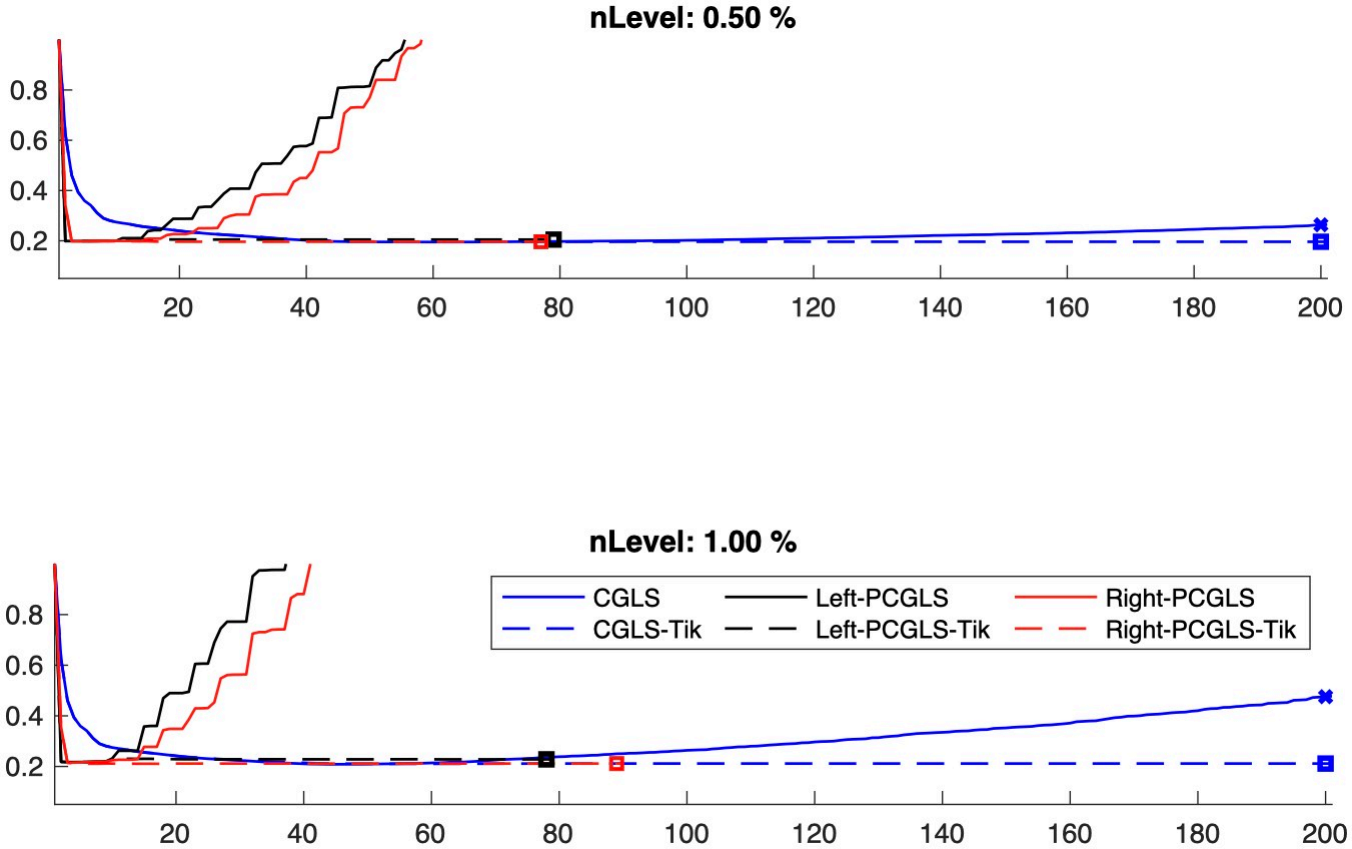
Results for preconditioned iterative methods. Relative reconstruction errors for preconditioned versus unpreconditioned iterative methods for Tikhonov regularization.

For Tikhonov regularization, we investigate the proposed preconditioners. We provide relative reconstruction error norms per iteration in Fig 7 for two noise levels 0.5% and 1%. In practice the noise level is usually much lower. We show that both left and right preconditioning result in faster convergence than unpreconditioned iterative methods. Furthermore although the preconditioned methods show semi-convergence behavior, whereby the error norms increase with later iterations, we can include appropriate regularization and compute a regularized solution. Thus, if one wishes to solve a large-scale nonlinear problem, preconditioned iterative methods can be used in an inner iteration to improve the overall efficiency of the algorithm.

### Nonlinear respirometry reconstruction

For the blind respirometry reconstruction problem where we assume the impulse response function is unknown, we investigate the performance of the described alternating optimization approach. For Algorithm 2, we need an initial guess of the impulse response function. For this we take a uniform function on the support of the impulse response function (see Fig 4) where the value is selected so that the impulse response function sums to 1. We found that our approaches can work for various choices of the initial guess of the impulse response function; however, it can be difficult to assess sensitivity for large-scale, nonlinear inverse problems. Our observation is motivated by our numerical experience in testing different initializations.

Recall that one of the nice features of the alternating optimization approach described in Algorithm 2 is that we split the main computational costs. Given an estimate of $\tilde{h}$, existing solvers can be used to compute a reconstruction $\tilde{x}$, and given an estimate of $\tilde{x}$, gradient-based constrained optimization methods can be used to efficiently estimate $\tilde{h}$. As we illustrated in Fig 3, the main challenge of the blind respirometry problem is the existence of multiple minimizers. In the experiments, we observe that using a 2-norm regularizer for $\tilde{x}$ resulted in overall smaller relative reconstruction error norms but much slower convergence. On the other hand, using a 1-norm regularizer for $\tilde{x}$ with FISTA was effective in avoiding the problem of getting stuck in undesirable local minimizers, especially at early iterations, but we needed to tune the regularization parameter. Here we use λ_1_ = 0.002. For estimating $\tilde{h}$, we use MATLAB’s lsqlin function to perform constrained optimization, where we enforce nonnegativity and summation to 1. For the choice of regularization parameter for $\tilde{h}$, we selected $\lambda_{\tilde{h}}=0.01$. We tried a range of values from 0.2 to 0.005, and as expected, the reconstructed impulse response function was smoother for larger values of $\lambda_{\tilde{h}}$.

**Fig 8:**
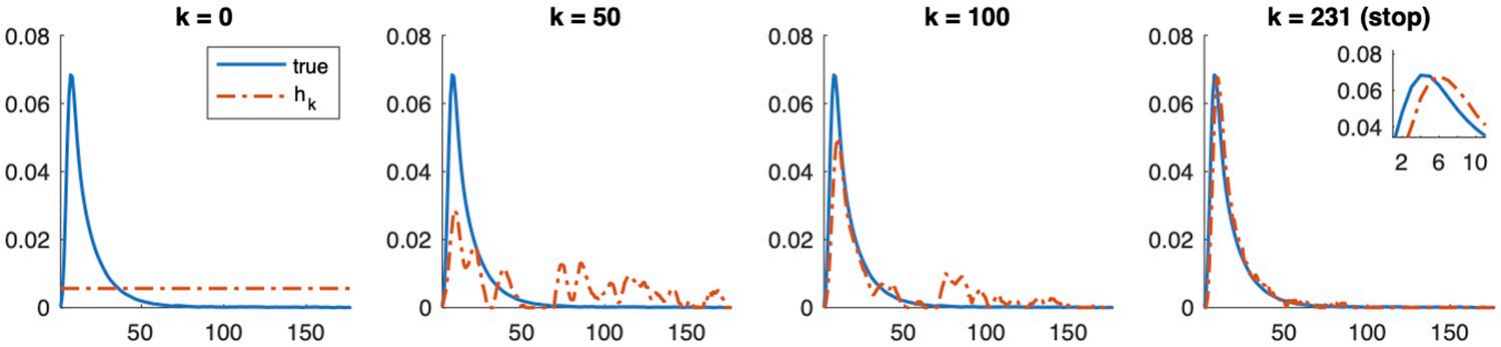
Reconstructed impulse response functions $\tilde{h}$ for the nonlinear respirometry problem at various iterations of the alternating optimization method. The subfigure in the right plot is a zoom of the peak of the reconstruction.

In Fig 8, we provide reconstructions of the impulse response function $\tilde{h}$ at various iterations of the alternating optimization method. Notice that the reconstructed function is shifted a few time units but is close to the support of the true impulse response function with small errors at the tails. Even though the initial guess is not close to true impulse response function, we obtain good reconstructions for the nonlinear respirometry problem.

**Fig 9:**
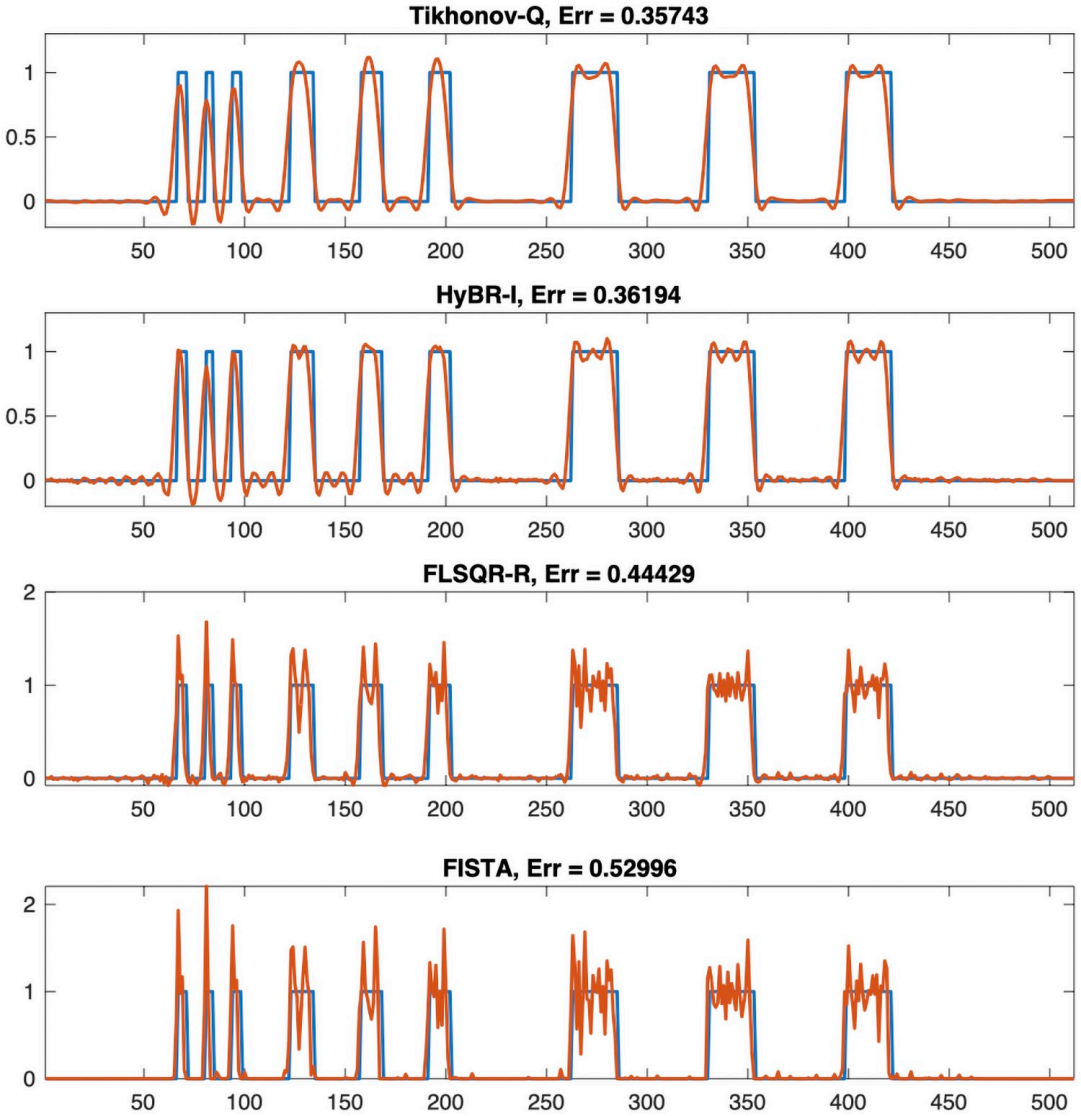
Reconstructed signals $\tilde{x}$ using different regularization techniques. All reconstructions correspond to $\tilde{h}$ in Fig 8.

**Fig 10:**
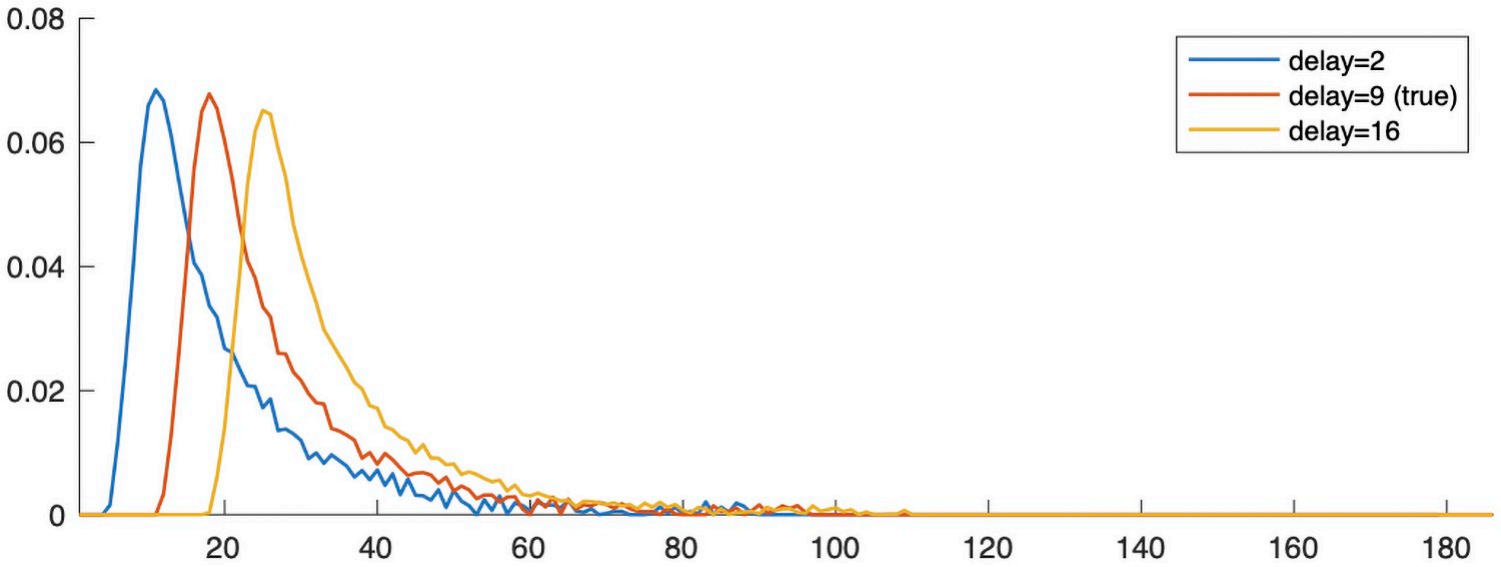
Investigation into the impact of selecting a different delay of the impulse response function in the nonlinear respirometry problem. Reconstructed impulse response functions $\tilde{h}$ are provided for different delays.

After obtaining a good reconstruction of $\tilde{h}$, we test various reconstruction methods to compute $\tilde{x}$. These results are provided in Fig 9 and show that FISTA and FLSQR-R are more sensitive than other methods when the reconstructed **h** has small errors at the tails. The regularization parameter was determined automatically in HyBR-I and FLSQR-R, while the optimal regularization parameter was used for Tikhonov-**Q**. Since the reconstructed $\tilde{h}$ is shifted, the relative errors are not small. However, we can observe that the shape of the reconstructed $\tilde{x}$ is close to the true $\tilde{x}$. Next, we investigate robustness of the proposed algorithm to an inexact delay. For real experiments, it is hard to know the exact delay of the impulse response function, and thus we must estimate it. As shown in Fig 10, our nonlinear optimization approach can still reconstruct an impulse response function whose shape is similar to the true function.

### Experimental validation

Finally, we test the described methods using real data from a flow-through respirometry chamber. First, we consider a linear reconstruction problem where we perfuse CO_2_ with an arbitrary pattern into a respirometry chamber and record the output concentration of CO_2_ with a gas analyzer. Then we apply the regularization methods (see Materials and methods) to reconstruct the exact CO_2_ injection pattern (i.e., the input signal) from the collected CO_2_ observations. For this example, we use a fixed experimental impulse response function and an empty chamber. Then to demonstrate the effectiveness of the nonlinear reconstruction methods on a real biological system, we include an insect in the chamber and use Algorithm 2 to obtain a joint reconstruction of the impulse response function and CO_2_ signal from the recorded CO_2_ observations. We compare the reconstructed instantaneous signal with abdominal movements of the living organism. From these two experiments, we show the performance of the proposed method in experimental respirometry inverse problems.

#### Linear case study

**Fig 11:**
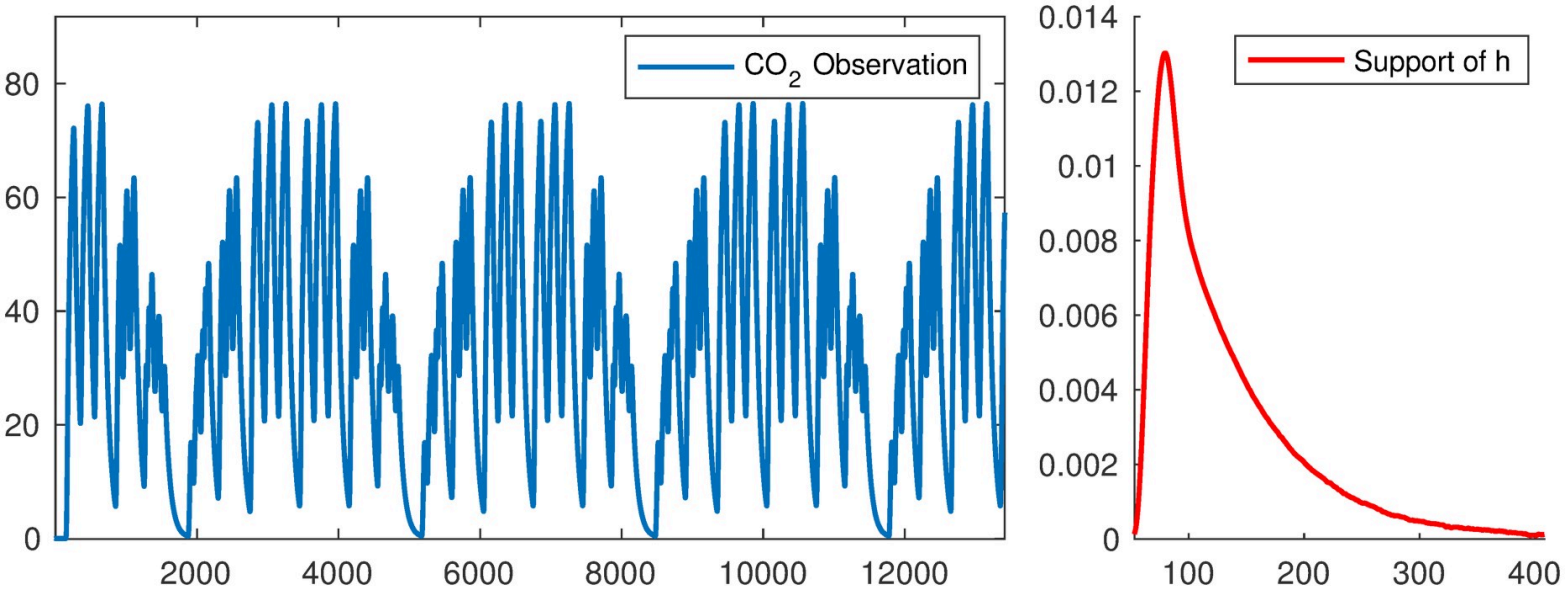
Experimental set-up for linear case study. CO_2_ observations from manipulated input CO_2_ signals to the empty chamber (left) and experimental impulse response function (right).

**Fig 12:**
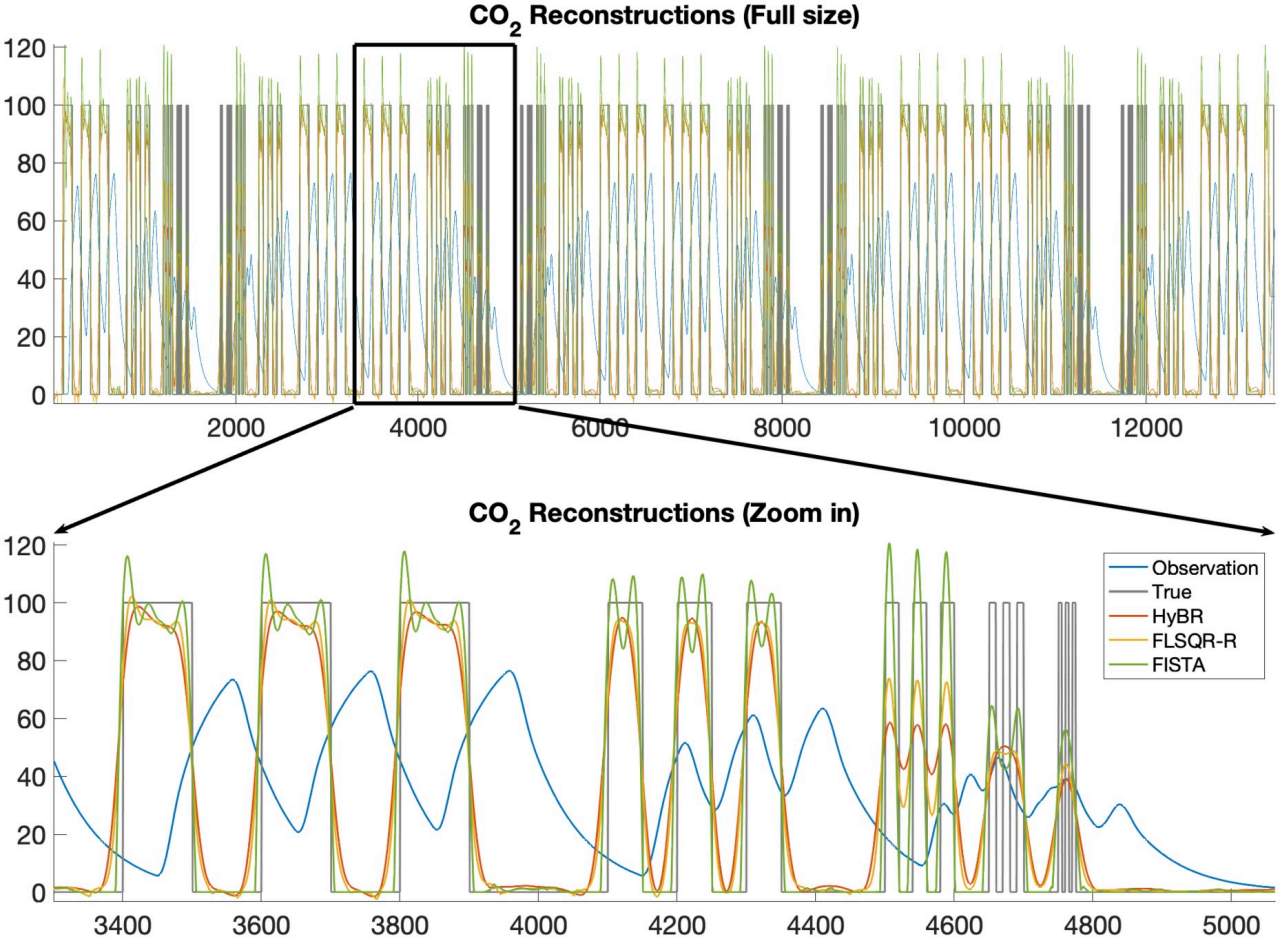
Experimental results for linear case study. Reconstruction of input CO_2_ signal using different reconstruction methods. A zoomed plot is provided in the bottom plot.

To validate the described methods, we designed an experimental setup to perfuse CO_2_ with a controlled pattern into a 28 ml (25 × 25 × 45 mm^3^) respirometry chamber. We used a high-speed valve (MHE2- MS1H-5/2-M7-K, Festo, NY, USA) to switch between dry air and CO_2_ gas (100 ppm, balanced with N2) immediately before the chamber. The inlet flow rate into the chamber was 250 ml/min. The outlet of the chamber was connected to an infrared gas analyzer (LI 7000 Li-Cor, Nebraska, USA). To test the accuracy of the methods, CO_2_ pulses with various width and frequencies were injected into the chamber and the concentration of the CO_2_ in the outlet was recorded with a sampling rate of 10 Hz. To determine the impulse response of the respirometry chamber, a short pulse of CO_2_ with the duration of 0.2s was injected into the chamber and the data were recorded for 5 minutes. The details of the experimental setup are described in Pendar et al [4, 5]. The output, observed CO_2_ signal and the experimental impulse response are provided in Fig 11. The size of the input and observed signal is 13, 413. For the linear reconstruction problem, we evaluate the following methods: HyBR-I, FLSQR-R, and FISTA. For HyBR-I and FLSQR-R, the regularization parameter is computed automatically using weighted GCV, and for FISTA, we use λ_1_ = 0.002. Reconstructions (including a zoomed image) are provided in Fig 12, along with the true signal and observation for comparison. Since the experimental impulse response function is well estimated in this case, all of the considered methods are able to nicely reconstruct the input signal. Notice that FISTA reconstructions are better able to resolve the peaks, especially when they are close together, as well as the flat regions (where no input is made).

#### Nonlinear case study: Abdominal pumping and CO_2_ emission in a darkling beetle

Next, we include a breathing insect in the flow-through respirometry chamber and investigate the performance of the blind respirometry reconstruction methods for simultaneously estimating the impulse response function and the CO_2_ instantaneous signal. A complicated network of tubes, called tracheae, run through an insect’s body to deliver oxygen to the tissues and return CO_2_ from the cells to the ambient air. The tube network is open to the outside air through valves which are called spiracles. Gas transport inside the tracheae occurs via diffusion and in larger insects via active ventilation, which is the result of compression of the tracheal tubes [41]. For larger and more active insects with higher metabolic rates, the diffusion is not sufficient to deliver enough oxygen to their tissues. They require an active ventilation to augment diffusive gas exchange. Active ventilation is known to be generated by abdominal pumping, a dorsoventral or anteroposterior compression of the abdomen [39, 42, 43]. However, some studies have shown that not all abdominal compressions are correlated with gas exchange, particularly in pupae and sub-adults [39, 44, 45]. In this study we used an adult tenebrionid beetle, Zophobas morio Fabricius, 1776 (Coleoptera: Tenebrionidae), to investigate the correlation between abdominal pumping and CO2 emission. Before putting the beetle inside the respirometry chamber, the beetle was cold-anesthetized at 3°C. Then its legs, head, and antennae were secured using adhesive putty (Scotch adhesive putty, 3M, Minnesota, USA) to prevent body movements during the recording. To see the abdominal movement the elytra and the soft wings were pinned to the sides.

**Fig 13:**
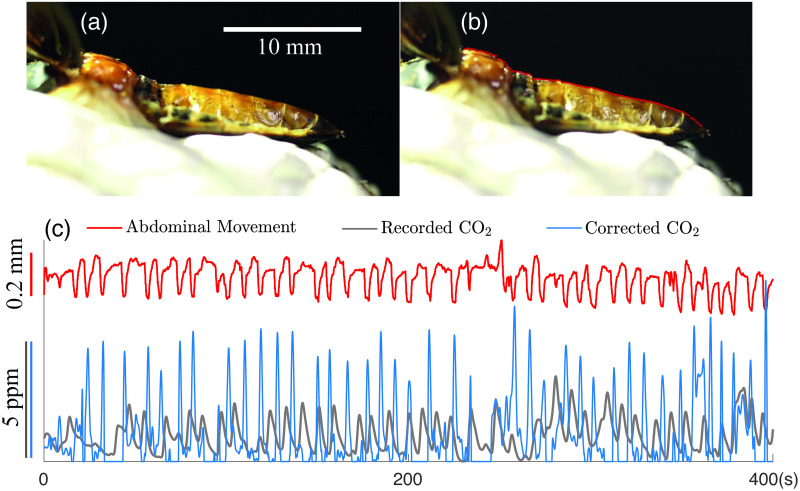
Experimental results for nonlinear case study. In this experiment, abdominal pumping and CO_2_ emission of a breathing insect are recorded and synchronized. (a) A darkling beetle is breathing in the respirometry chamber. (b) Abdominal movements of a darkling beetle (red dots) are recorded. (c) The abdominal movement (red) signal samples. The recorded CO_2_ emission samples (grey) from the chamber. The reconstructed CO_2_ signals (blue) with Algorithm 2 from a flat initial guess of the impulse response function, delay of 21.3 sec, and support of 18.4 sec. The recovered CO_2_ signal is concurrent with the abdominal movement of the insect.

After putting the secured beetle shown in Fig 13(a) inside the respirometry chamber and letting the beetle rest for an hour, we recorded CO_2_ emission and abdominal movement simultaneously. Recorded CO_2_ observations can be found in Fig 13(c). We also recorded the movement of the abdomen from the side with a video camera (NEX-VG10, Sony) at 30 frames per second. A flashing LED light was used to synchronize the video with the CO_2_ data. To process the recorded video we used a custom MATLAB code to track 120 equally spaced points along the mid-tergites (see the red points in Fig 13(b)) and considered the average displacement of these point as the dorso-ventral displacement of the abdomen.

**Fig 14:**
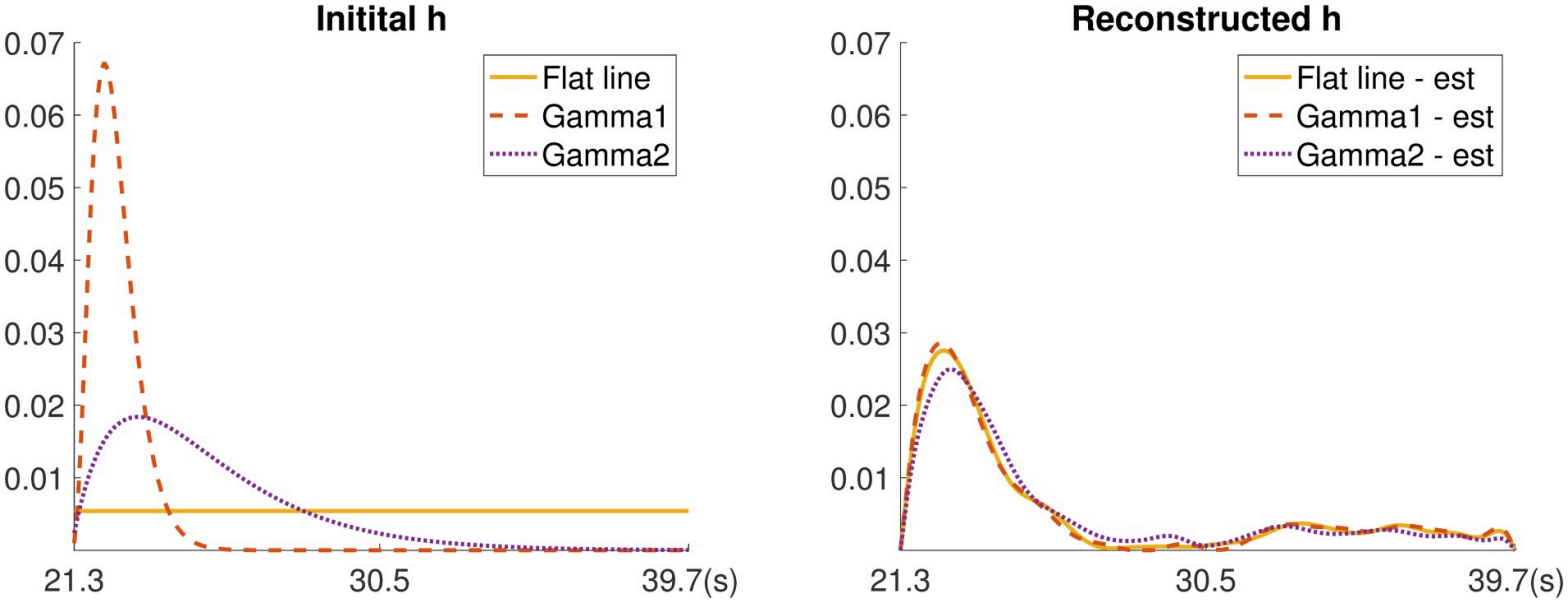
Impulse response function reconstruction for nonlinear case study. Initial guess for the unknown impulse response function (left) and reconstructed impulse response functions using a nonlinear respirometry reconstruction algorithm (right).

Then we used Algorithm 2 with the same regularization parameters used for the simulated experiments to simultaneously reconstruct the instantaneous CO_2_ signal from the observations and the impulse response function. We tested different initial guesses for the impulse response function ${\tilde{h}}_{0}$, where the support is 18.4 sec and the delay is 21.3 sec. First, following the work in [5], we considered density functions of Gamma distributions (e.g., $f\left(x\right)=\frac{\beta^{\alpha }}{\Gamma (\alpha )}x^{\alpha -1}\exp^{-\beta x}$) for different choices of *α* and *β*. Gamma1 corresponds to an initialization with *α* = 4 and *β* = 3, and Gamma2 corresponds to an initialization with *α* = 2 and *β* = 0.5. The initializations of the impulse response functions are provided in Fig 14. To investigate the sensitivity of our approach with respect to the initialization of ${\tilde{h}}_{0}$, we also considered an initial guesses where ${\tilde{h}}_{0}$ is a constant function. For each of the very different initial impulse response functions, the blind respirometry reconstruction method converges to an impulse response function with a similar shape and to similar reconstructed CO_2_ signals. The reconstructed impulse response functions are provided in the right panel of Fig 14. Notice that all functions must satisfy two conditions: nonnegativity and the area under the curve over the support is 1. The reconstructed CO_2_ signal (corresponding to an initialization of the flat line impulse response function) is provided in Fig 13(c). Since this is a real data experiment, we do not have the true signal to compare to. Thus, we verify our results by comparing the reconstructed CO_2_ signal to the recorded abdominal movement. In Fig 13(c), we provide a superposition of signals in order to show a correlation between abdominal movement and CO2 release.

## Conclusion

Respirometry reconstruction and especially blind respirometry reconstruction are crucial in interpreting the results of respirometry experiments in many physiological studies. We developed and investigated various computational tools for accurately and efficiently estimating the input signal, as well as the impulse response function, in any physiological system. By reformulating the linear respirometry problem in a Bayesian framework, we enabled tools for uncertainty quantification for both 2-norm and 1-norm regularization. Then, to accelerate the linear solve within iterative optimization methods (e.g., alternating optimization or Gauss-Newton methods), we developed preconditioners that are tailored to the respirometry forward model and demonstrated the excellent performance of these preconditioners for accelerating iterative reconstruction methods. Furthermore, by combining various constraints on both the impulse response function and the signal reconstruction, we developed sophisticated numerical optimization methods to tackle the very challenging problem of blind respirometry. Simulated and real-data results with a breathing insect demonstrate that these methods can be used to extract high temporal information for the original signal. Overall, these improvements in input estimation have the potential to change the way physiologists view indirectly recorded data, most particularly for studies of gas exchange, and can change the interpretation of the underlying physiological processes. To assist a researcher in implementing these methods for their own studies and to encourage further development of the methods, MATLAB code and data can be found at the website: https://github.com/T-Cho-vt/respirometry. Future work will be to develop efficient methods for uncertainty quantification that can exploit the separable nonlinear structure of the respirometry problem. Furthermore, we addressed efficient computation of variance estimates, but sampling methods for both the Gaussian approximation and the fully nonlinear problem are still open problems, especially for very large problems.
